# Comparison of incidence/risk of venous thromboembolism (VTE) among selected clinical and hereditary risk markers: A community-based cohort study

**DOI:** 10.1186/1477-9560-3-8

**Published:** 2005-07-20

**Authors:** Michael Spannagl, Lothar AJ Heinemann, Thai DoMinh, Anita Assmann, Wolfgang Schramm, Rolf Schürmann

**Affiliations:** 1 Ludwig-Maximillian-University Munich, Klinikum der Universität, Abteilung Haemostasiologe, Ziemssenstr.1, 80336 Muenchen, Germany; 2 Centre for Epidemiology & Health Research Berlin, Invalidenstr.115, 10115 Berlin, Germany; 3 Schering AG, SBU Fertility Control/Hormone Therapy, 13342 Berlin, Germany

**Keywords:** Cohort study, incidence, relative risk, inherited and acquired risk factors, venous thromboembolism.

## Abstract

**Background:**

Little information is available from community-based long-term VTE cohort studies to compare the absolute thrombosis risk of established clinical and genetic risk factors.

**Materials and methods:**

The occurrence of venous thromboembolism (VTE) was observed during a 10-year observation period in the BAvarian ThromboEmbolic Risk (BATER) study, a cohort study of 4337 women (age 18–55 years). We collected data on demographics, reproductive life, lifestyle, conditions/diseases, and particularly potential risk factors for VTE with a self-administered questionnaire. The objective was to present incidence rates of VTE and to show relative risk estimated associated with different clinical and genetic risk factors.

**Results:**

34 new, by diagnostic means confirmed VTE events occurred during the observation time of 32,656 women-years (WY). The overall incidence of VTE was 10.4 per 10^4 ^WY. The incidence rates varied markedly among different risk cohorts. The highest incidence was observed in women with previous history of VTE, followed by family history of VTE. None of the measured "genetically-related risk markers" (antithrombin, protein C, FVL, prothrombin mutation, or MTHFR) showed a significant VTE risk.

**Conclusion:**

Most of the discussed VTE risk factors showed no significant association with the occurrence of new VTEs due to smallness of numbers. Only first-degree family history of VTE and own history of a previous VTE event depicted a significant association with future VTE. Clinical information seems to be more important to determine future VTE risk than genetically related laboratory tests.

## Background

Long-term, community-based cohort studies designed to evaluate or compare both the risk of inherited and acquired risk factors for venous thromboembolism (VTE) in young women are lacking, only one Danish cohort study designed and conducted to examine cardiovascular risk factors and the relation with factor V Leiden (DNA was obtained later during follow-up) [1].

Information is rare on comparative differences in incidence rates and risk estimates across different clinical and inherited VTE risk factors generated in a community, i.e. making head-to-head comparisons possible within one study.

A community-based thromboembolic risk factor study started in the mid-1990s in Bavaria, the *BA*varian *T*hrombo*E*mbolic *R*isk study (BATER), focused on women in the reproductive age [2-4]. Clinical and hereditary risk markers for VTE, the lifetime history of relevant conditions or medications, and the family history of cardiovascular diseases were documented from 1993 throughout the follow-up period until 2003, i.e., carefully reviewing complaints or findings possibly related to the occurrence of venous clots.

The aim of this paper is to present risk estimates for VTE risk markers available in one study, and to provide incidence rates associated with these risk factors based on new VTE cases observed during follow-up.

## Methods

Material and methods of this long-term cohort study has been described in detail in earlier publications [2,3] and particularly in a recent publication in this journal [4]. In brief, we examined a cohort of 4337 young women (18–55 years) living in Bavaria who had at least one follow-up.

Data on demography, reproductive life, conditions/diseases, and particularly potential risk factors for VTE were collected through a self-administered questionnaire and subsequent telephone enquiries – if necessary- to supplement, clarify and verify the information in the questionnaires to set up the year 1993 as common starting point for all cohort members.

The source for the data on new (incident) VTE cases was the follow-up questionnaire (self-reported VTE or symptoms potentially compatible with VTE) completed by the study participants. This information was complemented by telephone interviews with the woman and with the treating physician. An external medical reviewer assigned all suspected VTE cases to one of five categories following an a priori defined decision scheme: *DEFINITE *(confirmation by imaging test), *PROBABLE *(unequivocal imaging test, other confirmatory tests positive, and anticoagulant therapy), *POSSIBLE *(unequivocal imaging test, suspicion in other tests, but no anticoagulant therapy), *POTENTIAL *(only clinical diagnosis without additional diagnostics, and no anticoagulant therapy), and *NO VTE *(alternative diagnosis). Details were given in a recent publication in this journal [4].

Possible and potential VTE cases were excluded from the analyses in this paper because of diagnostic uncertainty.

Women with a history of cancer, with chronic liver diseases, or with known antiphospolipid syndrome were not present in the study.

After having given informed consent the women included in this study gave a blood sample at entry into the study. An independent ethics committee approved all study related activities.

Whole blood samples were obtained from resting subjects. Blood was put into tubes with trisodium citrate. Plasma was prepared soon after venipuncture by centrifugation for 15 minutes with 3000 to 4000 rpm at room temperature and stored at - 20°C.

Protein C and antithrombin activities in plasma were measured by chromogenic substrate assays (Dade Behring, Marburg, Germany). For antithrombin (AT) the activity against factor IIa was determined, Protein C activity was measured after activation of the proenzyme by snake venom. Plasma activities are given as percentage (% of normal) of pooled human normal plasma.

Protein C deficiency was defined as less than 77% of normal (5^th ^percentile of the non-cases of the cohort). Antithrombin deficiency was assumed if less than 81% of normal (5^th ^percentile)

Genomic DNA was isolated by mean of QIAmp^® ^DNA Blood Kit (Qiagen) according to the manufacturer's instructions. The genetic polymorphisms Factor V R506Q (G1691A), Prothrombin G2010A and 5-, 10-methylenetetrahydrofolate reductase (MTHFR) A223V (C677T) were determined using a multiplex PCR with allele-specific primers slightly modifying a previously described method [5].

All blood tests were performed in a blinded manner, i.e. the investigators had no clinical information, nor access to the clinical database.

The database was structured to accommodate both concurrent as well as time-dependent variables. Concurrent variables are variables, which describe the woman's status at the time of questionnaire response, whereas the outcome variable is time-dependent. While concurrent variables were held in a fixed dataset, a periodic dataset containing information on the occurrence of VTE events along a time axis was created for each participant, using months as a unit of measurement. In this dataset, all exposures of interest in this paper, such as VTE risk factors including genetic markers, refer to the baseline point.

Some of the variables of interest (age, BMI, Protein C, AT) were continuous. These variables were dichotomized in order to define a categorical exposure status (exposed – non-exposed) for the analyses based on incidence or logistic regression. We arbitrarily separated the continuum in two roughly equal intervals such as age under/over 30 or BMI under/over 25 (kg/m2) in order to have sufficient cases for analyses with further stratification. For protein C and AT we used the lower 5th percentile (of the distribution in non-cases) as cut off point. This limit was considered as usual definition for "deficiency" and therefore clinically relevant [6].

Simple descriptive tables were prepared. All analyses concerning the occurrence of VTE events over time were performed by adding up individual observation time (1993 until the last contact) for different exposure cohorts and in total.

Apart from the overall VTE incidence rate per 10,000 women-years of observation (WY), we calculated also incidence rate ratios to compare the incidence of different sub-groups, e.g., women with factor V Leiden (FVL) mutation compared with women without this genetic mutation.

The calculation of the relative risk of occurrence of VTE is based on logistic regression analysis. Crude and adjusted odds ratios (OR) are reported with 95 % confidence intervals (95% CI).

All analyses were performed with the statistical packages SAS 8.2 or STATA 8.2.

## Results

The overall cohort encompasses 4337 women with sufficient information in 1993 and one follow-up at minimum. The observational period for our current analysis was 32,656 WYs since 1993.

Initially, 6082 eligible women were invited to join the study. Of these, 4372 (71.9%) agreed to participate in the follow-up and with the blood sampling. The main reason for non-participation was blood sampling.

The follow-up was carried out until 2003 at most, or otherwise terminated at the time when the last contact was possible to get information about new conditions that may have had occurred. 2076 women could be followed up until 2002/3 (47.9 %), 595 (13.7%) women did not participate in follow-ups before 1999–2001, and the largest proportion of women dropped out during the first years before 1999 (38.4%). Thus, the follow-up period was censored some time before 2002/3 for approximately half of the cohort members, i.e. the last successful contact was defined as "end of follow-up".

Thirty-four new cases of VTE occurred in the observational period. These cases were finally confirmed and categorized according to diagnostic certainty by an independent medical reviewer as definite (n = 31) or probable (n = 3). Cases with possible/potential VTE (n = 17) were excluded from further analyses because of low diagnostic certainty, i.e. it was not clear whether to classify them in the group cases or non-cases.

Out of the 34 definite/probable VTE cases 18 cases (= 52.9%) were associated with "clinical causes for VTE" and 16 (= 47.1%) were so-called "idiopathic" VTEs. The following "clinical causes" were observed prior to occurrence of the new VTE: 4 with previous VTE, 3 pregnancy/puerperium, 4 after an accident, 2 after surgery, 3 immobilization, and 2 after long travel in sitting position.

Table 1 depicts the profile of relevant data available at baseline (1993) to get an impression of the group under follow-up.

**Table 1:** Distribution of clinically and genetically relevant variables in a cohort of women at baseline of the observational period 1993 – 2003. The total number of women in this analysis is 4320, i.e. excluding 17 women with a final diagnosis of a possible/potential VTE. Deviations from this number are due to missing information

Variables			
**Continuous variables**		n	Mean (SD)

Age (years)		4320	26.0 (8.6)
Life births, number		1910	1.7 (0.8)
BMI^§^		4309	23.3 (4.1)
Protein C (% of normal)		4315	102.4 (15.8)
AT (% of normal)		4316	98.4 (11.3)
			
**Categorical parameters**			Percent (%)
Own history of VTE	No	4279	99.0
	Yes	41	1.0
Family history	No	3840	88.9
	Yes	480	11.1
Age, alternative	<30	2843	65.8
	≥ 30	1477	34.2
Family history of varicous veins	No	2395	55.4
	Yes	1925	44.6
Family history of MI	No	3830	88.7
	Yes	490	11.3
BMI, alternative	<25	3218	74.7
	≥ 25	1091	25.3
Ever use of hormone replacement	No	4031	93.7
	Yes	270	6.3
Family history of stroke	No	4013	92.9
	Yes	307	7.1
Ever use of oral contraceptives	No	346	8.0
	Yes	3973	92.0
Education level: Abitur^&^	No	3119	73.2
	Yes	1139	26.8
Ever smoker	No	2022	46.8
	Yes	2296	53.2
			
**Laboratory & genetic markers**			
Factor V Leiden mutation^1^	No	4035	93.7
	Yes	271	6.3
Prothrombin mutation^1^	No	4088	96.6
	Yes	142	3.4
MTHFR^1^	No	1798	42.5
	Yes	2432	57.5
Protein C deficiency^#^	No	4117	95.4
	Yes	198	4.6
AT deficiency^#^	No	4106	95.1
	Yes	210	4.9

^1 ^Homozygote & heterozygote together

^§^

Body mass index (kg/m^2^)

^&^maturity for university

^# ^definition see methods

The mean age was 26 ± 8.6 years, however, for the dichotomized age variable we used as cut-off point 30 years resulting in strata that contained VTE cases in both age groups. The frequency of other data, family history (first degree relatives) of potentially relevant diseases, conditions and genetic lab parameters is provided in the table 1. Homo-and heterozygote carriers of mutation were analyzed together because of small numbers or homozygote carriers.

Table 2 presents the characteristics of the 34 VTE cases and the remaining "non-cases" in the cohort. Varying total numbers in the table are due to missing information particularly in non-cases and genetic characteristics.

**Table 2:** Description of the subgroup of VTE cases and non-cases regarding parameters considered in this study as potential VTE risk factors at baseline of the observation period 1993 – 2003 Only definite and probable VTEs were considered as cases in this table, i.e. possible & potential VTE were excluded. Definitions of variables see text.

	Non-cases		VTE cases		Total cohort	
	N	n (%)	N	n (%)	N	n (%)

**Demographic data**						
Age*: 30+ years	4286	1456 (34.0)	34	21 (61.8)	4320	1477 (34.2)
BMI*: 25+	4275	1076 (25.2)	34	15 (44.1)	4309	1091 (25.3)
OC use: yes, ever	4285	3941 (92.0)	34	32 (94.1)	4319	3973 (92.0)
Other hormones: yes, ever	4267	267 (6.3)	34	3 (8.8)	4301	270 (6.3)
Smoking: yes, ever	4284	2278 (53.2)	34	18 (52.9)	4318	2296 (53.2)
						
**Medical history**						
Personal history of VTE*: yes	4286	37 (0.9)	34	4 (11.8)	4320	41 (1.0)
Family history of VTE*: yes	4286	470 (11.0)	34	10 (29.4)	4320	480 (11.1)
Family history of varicose veins*: yes	4286	1902 (44.4)	34	23 (67.7)	4320	1925 (44.6)
Family history of MI*: yes	4286	482 (11.3)	34	8 (23.5)	4320	490 (11.3)
Family history of stroke: yes	4286	302 (7.1)	34	5 (14.7)	4320	307 (7.1)
						
**Laboratory & genetic markers**						
FVL mutation^§^: yes	4273	267 (6.3)	33	4 (12.1)	4306	271 (6.3)
Prothrombin mutation^§^: yes	4199	140 (3.3)	31	2 (6.5)	4230	142 (3.4)
Protein C deficiency ^#^: yes	4281	4087 (95.5)	34	30 (88.2)	4315	4117 (95.4)
AT deficiency ^#^: yes	4282	4073 (95.1)	34	33 (97.1)	4316	4106 (95.1)
MTHFR^§^: yes	4199	2415 (57.5)	31	17 (54.8)	4230	2432 (57.5)

^§ ^homo- and heterozygote together

* significant difference between VTE cases and non-cases (p < 0.05)

^# ^definition see methods

There were some remarkable differences between cases and non-cases that affected differences in VTE risk estimates in further analyses such as incidence rates, incidence rate ratios as well as relative risk estimates (see below): cases were found to be older, to have a higher proportion of elevated BMI, of history of previous VTE, of family history of VTE (first degree relatives). Family history of varicose veins, myocardial infarction, and stroke were included not as VTE risk factors, but conditions with a potential for misclassification by respondents (past history was not validated).

Although the frequency of known genetic risk factors for VTE (specifically FVL mutation and prothrombin mutation) seemed to be higher in cases than non-cases, this was not statistically significant (see comparisons below). Only 6 of the 34 women suffering from definite or probable VTE showed any established marker of thrombophilia in the laboratory screen. Two of 6 patients in this group had severe thrombophilia with the combination of Protein C deficiency (48 % activity) and heterozygous Factor V Leiden or a homozygous FVL mutation. The other 3 patients exhibited only one positive laboratory marker and were either heterozygous for FVL (n = 2) or prothrombin mutation (PTM, n = 1). The numbers however were too small for sub-analyses. 24 patients showed no detectable marker of thrombophilia, 2 out of these patients demonstrated a homozygous MTHFR mutation.

We observed 34 new definite/probable VTE cases within the 32,508 WYs of observation, i.e., an incidence rate of 10.4 per 10,000 WYs.

Table 3 shows incidence rates of VTE stratified by presence (= exposed) or absence (= non-exposed) of the variables considered as potential "risk factors" in this analysis. Marked differences of incidence rates were observed across the variables listed in table 3, i.e., comparing the incidence between exposed and non-exposed in each of the variables. Several of the 15 compared parameters showed significantly elevated incidence rate ratios (relative risk): some demographic variables (advanced age, elevated BMI), data of the medical history (history of previous VTE, family history of VTE or family history of varicose veins). But to our surprise none of the established genetic VTE risk markers showed a significantly increased VTE risk in this analysis. However, taking the risk estimates at face-value, three of the five markers (positive FVL, prothrombin mutation, and protein C) depicted a 2-fold increase in risk, although statistically not significant. These however were only crude comparisons, i.e. do not account for the simultaneous influence of any of the other VTE risk factors.

**Table 3:** Incidence rates for VTE (definite and probable) based on 4320 women and 32,508 WY of observation (1993 – 2003). Tabulation by parameters considered as potential VTE risk factors. Descriptive tabulation of events per 10,000 WY in the exposed and non-exposed group. Incidence rate ratio (IR) and 95% confidence interval (95% CI). "Exposed" was defined as the group where the risk was assumed to be higher.

	Exposed		Non-exposed		Overall		Exp. vs. Non-exposed
Exposed vs. non-exposed	WY	VTE incidence per 10^4 ^WY	WY	VTE incidence per 10^4 ^WY	WY	VTE incidence per 10^4 ^WY	IR (95% CI)

**Demographic data**							
Age: 30+ years vs. <30	12,346	17.0	20,162	6.5	32,508	10.5	2.6 (1.3–5.7)
BMI: 25+ vs. <25	8,764	17.1	23,696	8.0	32,460	10.5	2.1 (1.01–4.4)
OC use: ever vs never	30,388	10.5	2,116	9.5	32,504	10.5	1.1 (0.3–9.6)
Other hormones: ever vs. never	2,484	12.1	29,896	10.4	32,380	10.5	1.2 (0.2–3.7)
Smoking: yes, ever	16,878	10.7	15,615	10.2	32,493	10.5	1.04 (0.5–2.2)
							
**Medical history**							
Personal history of VTE: yes	318	125.8	32,190	9.3	32,508	10.5	13.5 (3.5–38.3)
Family history of VTE: yes	3,810	26.3	28,698	8.4	32,508	10,5	3.1 (1.3–6.8)
Family history of varicous veins: yes	14,764	15.6	17,744	6,2	32,508	10.5	2.5 (1.2–5.7)
Family history of MI: yes	3,919	20.4	28,589	9,1	32,508	10.5	2.2 (0.9–5.1)
Family history of stroke: yes	2,480	20.2	30,028	9,6	32,508	10.5	2.1 (0.6–5.5)
							
**Laboratory & genetic markers**							
FVL mutation^§^: yes	2,105	19.0	30,308	9.6	32,413	10.2	2.0 (0.5–5.7)
Prothrombin mutation^§^: yes	1,010	19.8	30,817	9.4	31,827	9.7	2.1 (0.24–8.3)
Protein C deficiency^#^: yes	1510	26.5	30,958	9.7	32,468	10.5	2.7 (0.7–7.8)
AT deficiency^#^: yes	1648	6.1	30,828	10.7	32,476	10.5	0.6 (0.01–3.4)
MTHFR^§^: yes	18,423	9.2	13,404	10.4	31,827	9.7	0.9 (0.4–1.9)

^§ ^homo- and heterozygote together

^# ^definition see methods

Similar to the evaluation of the relative risk estimates using the incidence rate ratio in the cohort approach, the evaluation with crude odds ratios showed an almost identical set of significant risk markers (table 4): higher age, elevated BMI, personal history of previous VTE, family history of VTE & varicose veins, but in addition also family history of myocardial infarction. None of the five genetic markers was significantly associated with the VTE risk in the crude risk assessment.

**Table 4:** Potential VTE risk factors and risk estimates for VTE (definite and probable) based on 4320 women. Comparative assessment with logistic regression analysis: Odds ratio (OR) and 95% confidence interval (95% CI)

	Non-cases		Cases		Crude OR (95% CI)	Adjusted* OR (95% CI)
	Non-exposed	Exposed	Non-exposed	Exposed		

**Demographic data**						
Age: 30+ years vs. <30	2830	1456	13	21	3.1 (1.6–6.3)	2.4 (1.1–5.3)
BMI: 25+ vs. <25	3199	1076	19	15	2.3 (1.2–4.6)	2.0 (0.9–4.1)
OC use: ever vs never	344	3941	2	32	1.4 (0.3–5.9)	1.3 (0.3–5.8)
Other hormones: ever vs. never	4000	267	31	3	1.4 (0.4–4.8)	0.86 (0.2–2.9)
Smoking: ever vs. never	2006	2278	16	18	1.0 (0.5–1.9)	0.8 (0.4–1.7)
						
**Medical history**						
Personal history of VTE: yes vs. no	4249	37	30	4	15.3 (5.1–45.9)	6.6 (1.8–24.6)
Family history of VTE: yes vs. no	3816	470	24	10	3.4 (1.6–7.1)	2.4 (1.0–5.4)
Family history of varicous veins: yes vs. no	2384	1902	11	23	2.6 (1.3–5.4)	1.9 (0.8–4.1)
Family history of MI: yes vs. no	3804	482	26	8	2.4 (1.1–5.4)	2.0 (0.8–4.6)
Family history of stroke: yes vs. no	3984	302	29	5	2.3 (0.9–5.9)	1.2 (0.4–3.5)
						
**Laboratory & genetic markers**						
FVL mutation^§^: yes	4006	267	29	4	2.1 (0.7–5.9)	2.0 (0.7–6.0)
Prothrombin mutation^§^: yes	4059	140	29	2	2.0 (0.5–8.5)	2.3 (0.5–10.0)
Protein C deficiency^#^: yes	4087	194	30	4	2.8 (0.98–8.0)	3.0 (0.9–10.4)
AT deficiency^#^:yes	4073	209	33	1	0.6 (0.1–4.3)	0.5 (0.1–3.5)
MTHFR^§^: yes	1784	2415	14	17	0.9 (0.4–1.8)	0.95 (0.5–1.97)

^§ ^homo- and heterozygote together

^# ^definition see methods

* adjusted for all other variables

When the risk assessment of all mentioned parameters underwent a fully adjusted analysis, i.e. controlling for all other respective variables, the overall findings approximately were the same, but only higher age, personal and family history of VTE increased significantly the risk of VTE within the 10-year period. None of the genetic markers had a statistically significant impact on VTE risk – even not after adjustment for other potential risk factors. However, the two measured mutations and protein C remained at an apparently elevated VTE risk level – although these results were not statistically significant.

Virtually identical risk estimates were observed when the Cox regression was used instead of the logistic regression (data not shown), although based again on small numbers of exposed cases. Instable risk estimates due to small numbers and many adjustment variables cannot be excluded.

No significant interaction terms were found in the analyses (data not shown).

## Discussion

To our knowledge, there are no long-term community-based cohort studies designed to evaluate or compare the risk of inherited or acquired risk factors for venous thromboembolism (VTE) in women under the age of 50 years, except for a Danish cohort study which, at least initially, targeted general at cardiovascular risk factors and not VTE specifically (DNA was obtained later during follow-up) [1]. Moreover, this study was specifically focused on factor V Leiden.

It was our aim to evaluate or compare the absolute risk and risk ratio of established clinical or genetic risk markers for VTE. In the past, most risk factor studies for VTE were restricted to clinically available markers such as age, BMI, previous VTE, family history, or acute factors (immobilization, surgery, accidents, pregnancy/puerperium, and hormonal contraceptive use) and based on clinical or cross-sectional, observational studies or analyses in administrative databases. Many observational studies or cohort studies in young women did not consider inherited factors (overview about incidence and risk factor studies in [7,8]). Cohort studies in the population rarely included or reported genetic markers for thrombophilia and acquired, lifestyle-related risk factors, except the Physicians Health Study for example – the latter however only for males over 40 years of age [9], or the above mentioned Danish cohort study [1].

Other studies with focus on markers for hereditary thrombophilia were performed in patients (e.g. in anticoagulant clinics), in relatives of carriers of genetic mutations but not in the general population [6,10-12]. Point estimates for thrombosis-free survival in carriers of major thrombophilic states are often restricted to the selected cohort of family members only (overview in Crowther [13]). Moreover, the evaluation of genetic markers often does not consider the impact of clinically available risk factors and the design was mainly restricted to clinical or case-control studies.

Thirty-four VTE cases, classified as definite or probable, occurred within this period, which is equivalent with about 10 per 10,000 WYs. At the first glance, this incidence seems to be high. However, this might be the result of the specifics of our study: We put great effort on the detection of potential cases and – even more important – we included all definite and probable cases, whereas most of the reported incidence rates in young women refer only to "confirmed" and so-called "idiopathic VTE", i.e. excluded all cases that occurred in temporal relationship to other potential reasons such as pregnancy/puerperium, surgery, or immobilization, for example. A similar overall incidence rate of 12.3 events/10^4 ^person-years was observed in the Danish cohort study [calculated from – 1 -], which however covers both gender and a higher mean age (45 years in the Danish study vs. 26 years in our study).

Idiopathic VTE, however, reflects only a smaller part of all confirmed VTE cases [14]. In our cohort study we found roughly 53 % so-called "idiopathic" (primary) VTE cases, and the other roughly 47% of cases had a previous VTE in their history, or pregnancy/puerperium, surgery, or other reasons for immobilization/long bed-rest shortly prior to the VTE event. Thus, the incidence of "idiopathic VTE" observed in this study can be estimated to be about 5 per 10,000 WY. This is in agreement with other reported incidence rates in the general population [1,7]. The incidence estimates for definite VTE ranges between 1 to 6 per 10^4 ^WY in non-users or oral contraceptives (OC) and 2 to 10 per 10^4 ^WYs in OC users. Older studies depicted almost-always higher incidence rates than more recently performed studies (see overview in [7]). A recent systematic review [8] came to a pooled incidence of definite VTE for the general population of 5 per 10,000 person years, similar in males and females, and found that around 40% of VTE cases were "idiopathic". We conclude that our data can be generalized for the female population of this region in the fertile age range. This conclusion is supported by results of a prospective, community-based cohort study [9] that found a VTE incidence rate of 2.7 "primary VTE cases" per 10^4 ^person-years (equal to idiopathic: no previous VTE history, no cancer, no surgery or trauma), however, in males aged 40–49 years.

The absolute risks (incidence rates) varied markedly among those exposed or non-exposed by genetic and acquired VTE risk factors in our cohort study (see table 3). The relatively small numbers of women exposed to genetic VTE markers (see also table 2) should be taken into account before drawing conclusions. Thus, we have to be worry about statistically robust results in most of the sub-groups.

The crude, not adjusted comparison between "exposed" (= risk factor present) and "non-exposed" (= risk factor absent) showed incidence ratios (OR) ranging from 0.6 (AT deficiency) to 13.5 (personal history of VTE). The highest VTE incidence rate was found for women with a history of previous VTE (125.8 VTE cases per 10^4 ^WY – compared with 9.3 in women without VTE history), i.e., a 13.5fold increased incidence rate ratio (see table 3). Other significant incidence rate ratios were observed for higher age, elevated BMI, family history of VTE (and for varicose veins).

After fully controlling for all other risk factors (logistic regression) only age, personal and family history of VTE remained significant risk factors (see table 4). A significant role of the family history of VTE has been reported in several studies [15-17]. It is surprising that in our study the aggregate variable "family history" was more important than the individual genetic or related markers.

The impact of individual genetic markers on VTE risk (FVL, PTM, MTHFR) was not statistically significant in the logistic regression analysis (table 4) – possibly also due to the small number of incident cases. This is also true for Protein C and AT deficiency, i.e. when using the lower 5^th ^percentile as usual definition for deficiency [18]. Even FVL mutation did not show significantly increased VTE risk, although the point estimate (OR = 2.0) – at face-value – is compatible with the estimate of another, but much larger cohort study [1], but lower than reported from several case-control studies. This difference between risk estimates for FVL in cohort and case-control studies is probably due to methodological reasons [1].

A similarly weak impact of genetic markers was also observed in follow-up studies of selected groups: Carriers of genetic polymorphisms have been followed prospectively and found a low absolute annual incidence of VTE [10,12,19]. Another prospective cohort observed a low incidence of VTE in otherwise healthy thrombophilic children [20] or asymptomatic family members who are carriers of factor V Leiden [11,12] or other family studies [21]. Deficiency of AT and PC activity had also no significant impact on thrombotic risk. We cannot exclude however that a considerable part of such unexpected results for PC and AT activity may be related to pre-analytical conditions in our field study. From a laboratory perspective in a few women polymorphisms leading to decreased levels of analytical results but not to clinical manifest thrombophilia are quite possible. An explanation might be that silent mutations and polymorphisms cause reduced activities in the laboratory assays not reflecting a potential clinical problem [22,23]. Without a positive personal or family history of VTE such results should at least be confirmed with additional laboratory tests and family examinations before informing the patients of a potential thrombophilic diathesis or before recommending respective medical prophylaxis.

There is an increasing debate about the role of genetic factors in the prediction of future VTEs and thus sustaining a controversy about genetic screening. This issue is a current controversy in the literature [24,25]. Clinical reports often suggest a high VTE recurrence rate in patients with previous VTE [26], but we found this phenomenon only in 4 of our 34 incident VTE cases. A recently published community-based cohort study of FVL carriers & non-carriers observed no significant difference on VTE incidence between both groups, except for women ≥ 60 years of age [27].

In general, our results support the notion that genetic parameters alone are relatively weak long-term risk factors; the occurrence of VTE requires interaction of both inherited and acquired risk factors or gene-gene-interactions [28].

The clinical VTE risk factors with significantly elevated incidence rate ratios such as advanced age and elevated BMI, but also history of previous VTE, family history of VTE or family history of varicose veins are not new. Most physicians are aware of these risk factors and consider them in practice. No incidence difference was also found for ever smoking which is rarely considered as risk factor for VTE. Hypertension was not analyzed. No significant prognostic impact was found for ever use of hormonal treatment/contraception at baseline. This is plausible because sex hormones effects are not general characteristics but short-term acting factors, which need another statistical approach, and was not the aim of this study.

The influence of more acutely acting risk factors – such as immobilization, surgery, long-haul flights, and use of drugs (e.g. OCs) was intentionally excluded from this analysis, although interactions between hereditary factors and acute, environmental pattern are known [21,29,30]. Only parameters that were available at baseline and likely to affect the long-term development were eligible for this analysis. Other influential risk factors or preventive measures have to be considered when discussing activities to reduce a predicted increased risk in the medical practice. It was not the aim of the study and data are neither available to test the effect of preventive measures nor the effect of additional risk factors in the immediate period before the event occurred. This would require another study design and a separate study with sufficient power for such questions.

It is a limitation of this long-term cohort study, however, that the number of incident, confirmed (definitive and probable) VTE cases was still small in absolute numbers (n = 34) in this cohort observation period of 32,508 years of observation. The low incidence can be explained by the young average age (26 years at entry). Thus, incidence & and risk estimates have wide confidence intervals and conclusions are limited. Rare combinations of risk markers have not yet materialized in one single VTE case. This makes it difficult to further divide into cases that occurred in presumably exposed or unexposed sub-groups. This is particularly true if the potential risk factors (exposure) are infrequent, as it is for genetic markers. It is a problem of the study design that no attempt was made to confirm of deficiencies (second blood sample) and no family study was planned to assess inheritance of deficiencies such as AT or PC.

In case of FVL mutation the adjusted risk is apparently increased about 2fold (non-significant) but only 4 cases had a positive test of FVL mutation (for prothrombin mutation only 2 cases). If one then adjusts for 14 other potential factors, there is obviously a statistical problem: Resulting risk estimates might be unstable, i.e. drifted in either direction. Even if one focuses on the crude OR, which is similar to the adjusted estimate in this case, careful interpretation is warranted. It cannot be excluded that the "true risk" of the mutation markers is 2fold increased, although the risk estimates do not favor of such a conclusion. Insofar, future analyses will benefit from an improved point of departure (longer observation, more cases).

## Conclusion

In conclusion, estimation of VTE risk cannot be based on genetic characteristics alone – but only in combinations with available clinical information. Genetic markers play obviously a limited role in the long-term prediction of VTE. Genetic markers form together with the "environmental circumstances" the disposition, i.e. together with the family history of cardiovascular events, specifically venous events. If the disposition translates into an event, this is obviously more influenced by "longstanding clinical VTE risk factors", factors such as positive medical history, advanced age, and elevated BMI than specific genetic factors. However, there are obviously other important, more acutely affecting environmental factors such as immobilization, surgery, and treatment with drugs that influence coagulation. The latter factors can be used to reduce the basic risk determined by long-term acting acquired risk factors modulated by inherited factors.
